# Gyroid as a novel approach to suppress vortex shedding and mitigate induced vibration

**DOI:** 10.1038/s41598-025-11199-0

**Published:** 2025-07-16

**Authors:** Thomas Berger, Mohamed Farhat

**Affiliations:** Institute of Mechanical Engineering, École Polytechnique Fédérale de Lausanne, Avenue de Cour 33 Bis, 1007 Lausanne, Switzerland

**Keywords:** Fluid dynamics, Mechanical engineering, Aerospace engineering, Applied physics

## Abstract

The present study uncovers how a Gyroid-structured extension, attached to a hydrofoil trailing edge, may prevent the formation of Karman vortices and remarkably reduce vortex-induced vibration (VIV). The case study is a blunt truncated NACA 0009 hydrofoil of 100 mm chord length and 150 mm span, placed in a water stream at high Reynolds number (Re = 0.6 × 10^6^ to 2 × 10^6^). In the absence of the Gyroid extension, as the flow velocity is increased from 6 to 20 m/s, the alternate Karman vortices generated in the wake are responsible for the hydrofoil vibration with a strong torsional lock-in at flow velocities ranging from 15 to 17 m/s. The Gyroid extension, however, largely reduces the flow-induced vibrations and the lock-in is completely suppressed. Specifically, the RMS value of the surface velocity signal is cut by 67% under lock-off conditions and 99.5% under lock-in conditions. Detailed velocity measurements in the wake using laser Doppler velocimeter confirm that the Gyroid insert eliminates the frequency peak associated with the Strouhal shedding frequency and reduces broadband noise excitation. These measurements uncover how the combination of porosity and tortuosity of the Gyroid insert prevents the formation of coherent and periodic Karman vortices. In particular, we found that the Gyroid extension is responsible for the generation of streamwise and transverse jets, which extend into the far wake, inhibiting the roll-up of transient vortices in a remarkable way. We believe that this is the key mechanism in suppressing vortex shedding. Interestingly, the measurement of lift and drag forces did not reveal any significant alteration of the hydrodynamic performances of the hydrofoil with the Gyroid extension. These promising results have far-reaching implications for the design of mechanical structures subjected to VIV, such as aircraft wings, marine propellers, hydraulic pumps, and turbines among others. The potential benefits include reduced noise emissions and mitigated fatigue risks.

## Introduction

In 1889, Engineers were able to overcome the wind static pressure, by building the iconic Eiffel Tower in Paris, which surpassed the 1000-foot (305 m) milestone as the tallest structure of its time. This remarkable achievement was made despite a limited understanding of wake dynamics and its interaction with a mechanical structure—concepts that would only be formally established two decades later by Theodore von Kármán^1,2^. The tower’s porosity, which Eiffel praised for reducing drag, likely played a crucial role in mitigating vortex shedding and preventing the formation of large coherent vortices.

It is now well established that beyond a critical Reynolds number, a fluid stream around a bluff body develops wake instabilities, leading to the shedding of alternating vortices known as *Kármán vortices*. The resulting oscillatory forces can induce mechanical vibrations and noise radiation, which may have severe consequences if resonance occurs. As first explored by Koopmann^3^ and Griffin^4^, when the shedding frequency approaches the structure’s eigenfrequency, the amplitude significantly increases, leading to resonance. Moreover, because of the high-amplitude vibrations, the flow, and the structure couple together and the shedding frequency may lock onto the natural frequency of the structure. This phenomenon, known as lock-in, is particularly dangerous because it can persist over a wide range of freestream velocities. Vortex-Induced Vibrations (VIV) are a critical concern in various engineering applications, such as offshore structures, underwater pipelines, marine risers, aircraft and vehicle components, turbomachinery, bridges, transmission lines, and tall buildings.

Over the years, countless flow control techniques have been proposed to mitigate VIV. Whether active or passive, these methods share a common objective: disrupting the formation of coherent and periodic vortices to prevent them from strengthening. The use of porous inserts to reduce noise and tip vortices was first introduced by Hayden, Smith, and Revell^5–7^. Advances in metal foam manufacturing prompted further investigations into porous airfoils by Geyer^8,9^ and, more recently, by several other researchers^10–14^. Rubio^15^ proposed porous inserts with a regular pattern, such as cylindrical holes, while Ayton^16^ developed bio-inspired porous inserts modeled after owl and buzzard wings. With the development of 3D-printing, more intricate lattice structures have been recently explored by Luesutthiviboon^17^ and Teruna^18^. These studies consistently demonstrated the effectiveness of porous inserts in reducing noise and vibration at low and medium frequencies. However, they also revealed the presence of spectral peaks at high frequencies.

In the present study, we investigated the use of a Gyroid-based trailing edge to mitigate vortex-induced vibration of a lifting surface. Gyroids, first discovered by Alan Schoen^19^ in 1970, are porous cellular-like structures that seamlessly repeat in all 3 dimensions, perfectly tiling the space to any arbitrary bounds. As part of the Triply Periodic Minimal Surfaces (TPMS), they locally minimize their area and exhibit a zero-mean curvature everywhere. Gyroids offer high strength-to-weight and surface-to-volume ratios. These unique properties made Gyroids a popular infill pattern in 3D-printing as well as in tissue engineering, acoustics, bone implants^20^, chemical engineering^21^ and heat transfer^22^. Naturally occurring Gyroids have also been observed in biological membranes^23^ and butterfly wing scales^24^. Here, we propose for the first time the use of Gyroids in flow control applications.

**Fig. 1:**
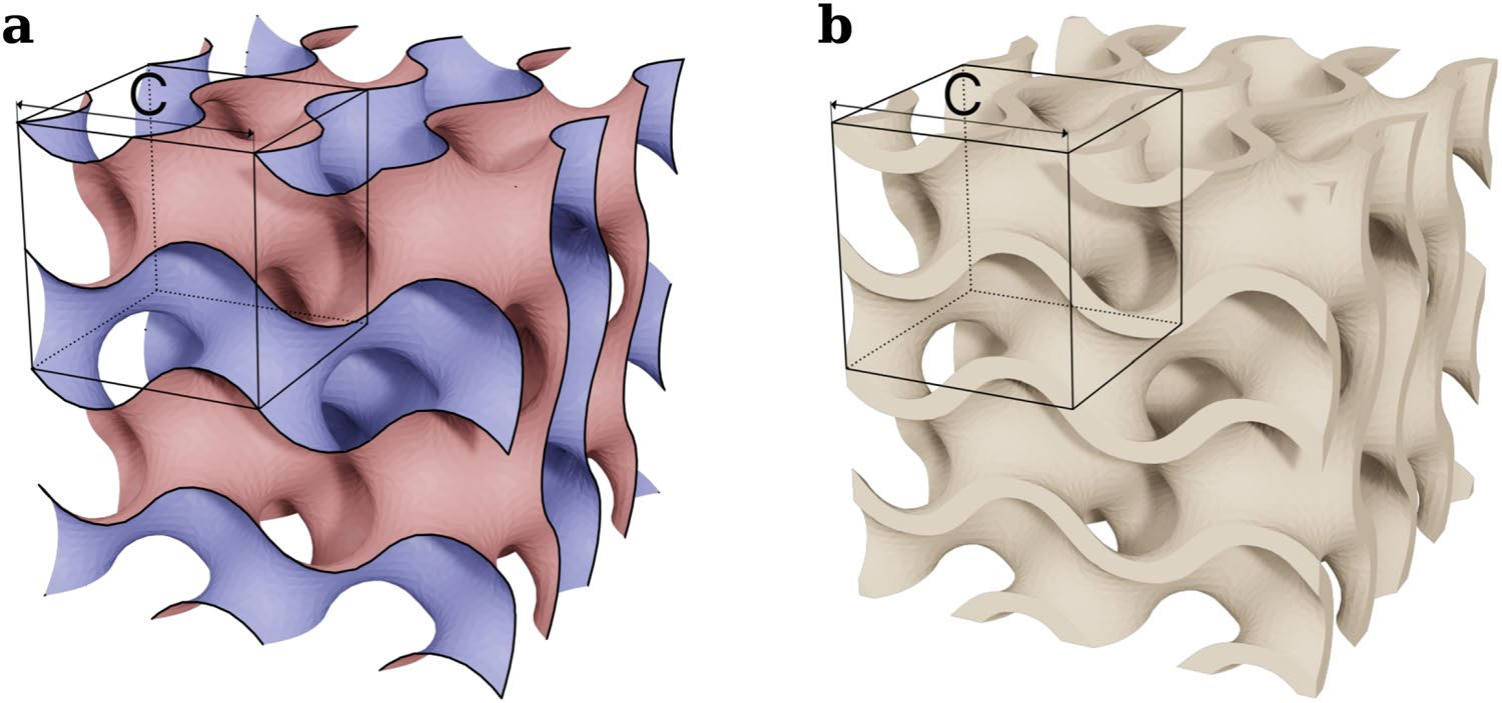
Illustration^28^ of 8 unit-cells of the gyroid, along with the unit-cell cube. (**a**) Gyroid surface colored with two surface sides. (**b**) Gyroid sheet-base volume.

Gyroids^19^ were originally created by geometric construction using rotations and reflections of a curved triangular fundamental patch. Later on, it was demonstrated that the gyroid can be closely approximated by a level-set surface satisfying the equation^25,26^:

$$\varphi \left(x,y,z\right)=\text{sin}\left(\lambda x\right)\text{cos}\left(\lambda y\right)+\text{sin}\left(\lambda y\right)\text{cos}\left(\lambda z\right)+\text{sin}\left(\lambda z\right)\text{cos}\left(\lambda x\right)=0$$

where *λ* = *2π/C* and *C* defines the cubic unit cell which corresponds to the spatial periodicity of the crystal structure. By introducing a thickness *t* around this zero level-set, a volumetric gyroid sheet-based structure can be obtained, making it suitable for 3D printing (Fig. 1). The gyroid has several interesting properties. Große-Brauckmann^27^ proved the gyroid’s embeddedness, which ensures that it can be smoothly represented in ${\mathbb{R}}^{3}$ without singularities or self-intersection. Additionally, the gyroid is orientable, meaning it can be globally assigned a continuous normal vector. It follows that the gyroid is a two-sided surface, which partitions the three-dimensional space into two distinct sub-volumes defined by *φ(x,y,z)* > *0* and *φ(x,y,z)* < *0*. These sub-volumes are intricately interwoven with each other making the gyroid unique. The two disjointed domains may be also seen as two networks of helicoidal pores with a diameter of *C/2-t* and a sweep amplitude of *C/4*. Despite being seamlessly interconnected, the pores in one domain do not connect with those in the other one. The two networks are intertwined in a remarkable way to form highly streamlined and permeable medium, yet with a high tortuosity.

We evaluated the performance of a Gyroid-based trailing edge in mitigating Vortex-Induced Vibration (VIV) for a NACA 0009 hydrofoil (100 mm chord length, 150 mm span) in a water tunnel. We conducted vibration measurements by gradually varying the upstream velocity over a wide range (6 to 20 m/s). Additionally, we performed flow velocity surveys in the wake using Laser Doppler Velocimetry (LDV). To assess the impact of the Gyroid insert on hydrodynamic performances, we used a hydrodynamic balance to measure lift and drag forces.

## Results

**Fig. 2:**
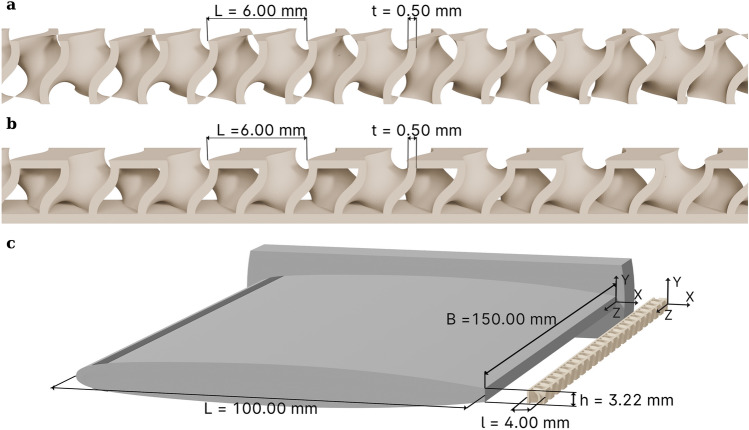
Modelling steps^28^ of the gyroid-based extension and foil setup with gyroid based extension. (**a**) Gyroid sheet-based structure. (**b**) The gyroid based extension with the bonding face at the proximal side and the seals on every second pore on the distal side. (**c**) NACA 0009 hydrofoil with a blunt trailing edge and the gyroid-structured extension.

The results presented here were obtained with a specific design of a gyroid-structured insert, attached to the blunt trailing edge of a baseline NACA 0009 hydrofoil, as illustrated on Fig. 2. The boundary layer is tripped at the leading edge with the help of a distributed roughness as described by Ausoni^29^. The Gyroid extension has a unit cell of 6 mm and a wall thickness of 0.5 mm, so that the mean size of the fluid passages is of the same order as the viscous core of the trailing vortices^30^. The proximal side of the insert was sealed with a thin plate to facilitate its bonding. The distal side was partially sealed to preserve the gyroid channeling property. The design steps are illustrated in Fig. 2. The coordinate system is set on the chord line, at the root of the hydrofoil, at the trailing edge. The *x, y* and *z* axes are oriented in the streamwise, transverse and spanwise directions, respectively. When the Gyroid insert is used, the coordinate system is shifted by 4 mm streamwise, such that it remains positioned on the trailing edge.

### Vibration analysis

We monitored the flow-induced vibrations of the hydrofoil at zero-incidence angle, both with and without the Gyroid insert. The flow velocity at the inlet of the test section was steadily and linearly increased from 6 to 20 m/s over a duration of 165 s. With such uniform and very low acceleration (0.085 m/s^2^), we can reasonably assume the flow is in a quasi-steady state. This ramped velocity approach not only expedited the experiments but also minimized the risk of missing any relevant transitions.

**Fig. 3:**
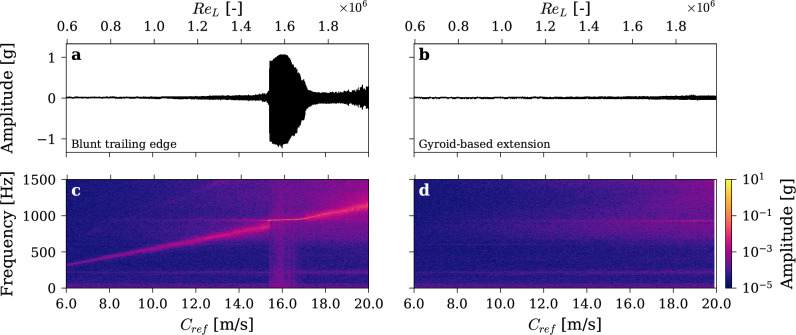
Vibration signals and their time–frequency analysis during velocity ramp-up tests with (**a**,**c**) Blunt trailing edge and (**b**,**d**) Gyroid-based extension.

Figure 3 presents the broadband vibration signals and their corresponding spectrograms as functions of the free-stream velocity. For the hydrofoil with a blunt trailing edge, shown in Fig. 3c, most of the spectral energy is concentrated around the vortex shedding frequency, as expected, adhering to the linear Strouhal relationship with *St ≈ 0.18*. The natural bending and torsional frequencies of the foil, *f*_*B*_* ≈ 220 Hz* and *f*_*T*_* ≈ 940 Hz*, are excited throughout the test and are clearly visible as horizontal bands. Notably, when the forcing frequency approaches the foil’s natural torsional frequency, resonance occurs, resulting in a significant increase in vibration amplitude, exceeding *1 g*. Consistent with Ausoni’s findings^31^, a lock-in phenomenon is observed between *Re* = *1.53 *× *10*^*6*^ and *Re* = *1.69 *× *10*^*6*^. During this phase, the flow and the foil form a hydro-elastic coupling, and the shedding frequency synchronizes with the foil’s torsional frequency over a wide velocity range. Upon exiting the lock-in regime, the shedding frequency gradually detaches and resumes following the Strouhal relationship.

The tests were repeated identically with the same hydrofoil equipped with a gyroid-shaped extension, attached to its trailing edge. The vibration measurements, presented in Fig. 3b, reveal that the hydrofoil remains almost silent with a significant reduction of flow-induced vibration throughout the upstream velocity range. In particular, the vibration amplitude does not exceed 5 × 10^*–2*^* g* and the hydrofoil resonance and lock-in are completely suppressed. As presented on Fig. 3d, while the natural frequencies of the hydrofoil remain weakly excited, no prominent shedding frequency can be identified.

**Fig. 4:**
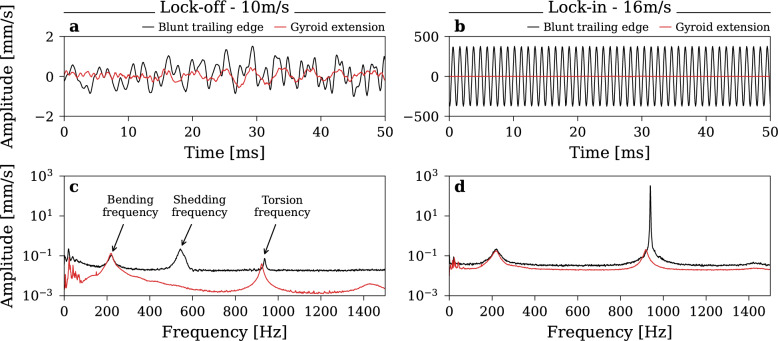
Vibration signals and their frequency analysis at steady velocity with the Blunt trailing edge and the Gyroid-based extension, at (**a**,**c**) C_ref_ = 10 m/s and (**b**,**d**) C_ref_ = 16 m/s.

The vibrations are also measured with a laser vibrometer directly on the hydrofoil surface under steady velocities at *C*_*ref*_ = *10 m/s* and *C*_*ref*_ = *16 m/s* (baseline lock-in conditions). The results are presented in Fig. 4. In both situations, the gyroid extension results in a significant reduction in vibration amplitude. Specifically, from *A*_*RMS*_ = *1.17 to 0.39 mm/s* (a 67% reduction) at *C*_*ref*_ = *10 m/s*, and from *A*_*RMS*_ = *268.81 to 1.27 mm/s* (a 99.5% reduction) at *C*_*ref*_ = *16 m/s*.

Frequency analysis confirms the complete elimination of the vibration response to the vortex excitation with the gyroid extension. At *C*_*ref*_ = *10 m/s*, the vortex shedding frequency *f*_*s*_ = *540 Hz* results in a vibration amplitude of *A* = *0.25 mm/s* for the blunt trailing edge. On the contrary, no discernible peak is observed around this frequency with the gyroid extension, indicating the effective suppression of vortex shedding excitation. Additionally, the overall noise level, outside resonance, is slightly lower with the gyroid extension compared to the blunt trailing edge.

When inspecting specific frequency peaks, the bending frequency exhibited similar characteristics with both trailing edge shapes. However, the torsional frequency peak demonstrates a substantial reduction in response amplitude with the gyroid extension. At *C*_*ref*_ = *10 m/s,* the response amplitude is reduced from *0.07 to 0.04 mm/s*, while at *C*_*ref*_ = *16 m/s*, the lock-in is eliminated, reducing the response from *325 to 0.20 mm/s*. Moreover, the torsional frequency with the gyroid extension appears slightly lower than with the blunt trailing edge, suggesting a potentially higher hydrodynamic damping.

### Where have Kármán vortices gone?

**Fig. 5:**
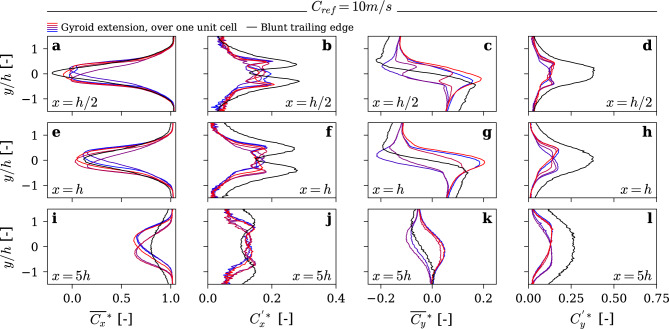
Velocity measurements at 3 streamwise locations at C_ref_ = 10 m/s. Presents (**a**,**e**,**i**) normalized mean streamwise velocity; (**b**,**f**,**j**) normalized streamwise velocity fluctuation; (**c**,**g**,**k**) normalized mean transverse velocity profiles; and (**d**,**h**,**l**) normalized transverse velocity fluctuation. Measurement stations are located at (**a**–**d**): x = h/2, (**e**–**h**) : x = h, and (**i**–**l**): x = 5 h.

**Fig. 6:**
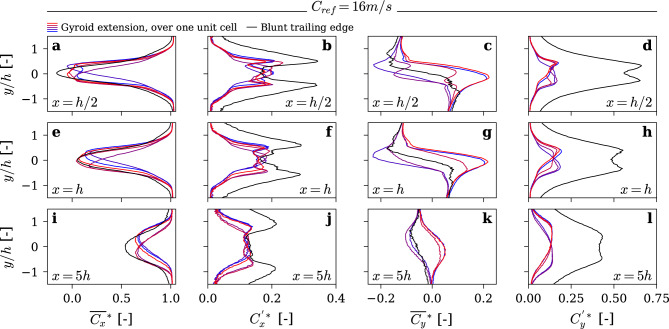
Velocity measurements at 3 streamwise locations locations at C_ref_ = 16m/s. Presents (**a**,**e**,**i**) normalized mean streamwise velocity; (**b**,**f**,**j**) normalized streamwise velocity fluctuation; (**c**,**g**,**k**) normalized mean transverse velocity profiles; and (**d**,**h**,**l**) normalized transverse velocity fluctuation. Measurement stations are located at (**a**–**d**): x = h/2, (**e**–**h**) : x = h, and (**i**–**l**): x = 5 h.

In order to better understand the reasons behind the spectacular vibration reduction, we investigated the wake flow using Laser Doppler Velocimetry (LDV). Streamwise and transverse components of the wake velocity along transverse lines were measured for both baseline and gyroid hydrofoils. The measurements are conducted at three locations along the streamwise direction at *x* = *0.5 h, x* = *h and x* = *5 h*. These stations were selected to capture the flow behavior within and immediately downstream of the vortex formation region, as identified by Ausoni^29^, as well as further downstream where the wake is fully developed. For the baseline hydrofoil, the spanwise location of the measurement was fixed at *z* = *131 mm*. For the gyroid hydrofoil, additional measurements are performed at five locations along the spanwise direction *(z* = *131, 132.5, 134, 135.5, 137 mm)*, covering one unit cell of the gyroid structure. Figure 5 and Fig. 6 present the mean and standard deviation of normalized streamwise and transverse velocities, at *C*_*ref*_ = *10 m/s* and *C*_*ref*_ = *16 m/s, respectively*.

For the blunt trailing edge, the wake exhibits a well-known behavior in lock-off conditions^29^, characterized by a strong velocity deficit that quickly dissipates downstream. The streamwise velocity fluctuations (C^*^_x_) follow a classical M-shape, while the transverse velocity fluctuations (C^*^_y_) form a bell shape. Under lock-in conditions (at *C*_*ref*_ = *16 m/s)*, the M-shape of C^*^_x_ becomes more pronounced, and the bell shape of C^*^_y_ transitions into an M-shape. These changes are the result of the hydro-elastic coupling, which generates more coherent and intense Kármán vortices.

Figures 5 and 6 illustrate a profound transformation in the wake structure induced by the Gyroid insert. Notably, the wake becomes considerably thinner, and the velocity profiles exhibit strong spanwise variations, with distinct velocity extrema corresponding to different locations within a Gyroid unit cell. Moreover, velocity fluctuations are significantly reduced, and the wake persists further downstream compared to the baseline hydrofoil. This clearly indicates lower turbulence levels and reduced mixing between the wake flow and the outer flow. Interestingly, the non-dimensional velocity profiles remain remarkably unchanged with the Gyroid insert when the upstream velocity is increased from 10 to 16 m/s. This is in line with the vibration measurements presented above, which highlight a total elimination of the lock-in with the Gyroid insert.

**Fig. 7:**
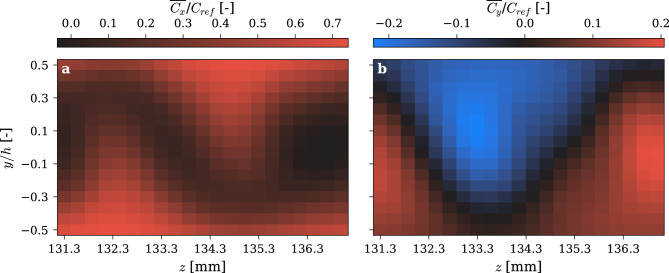
Spanwise-Transverse velocity measurement over one gyroid unit cell in the wake of the hydrofoil with Gyroid-based extension at Cref = 10 m/s. (**a**) Streamwise velocity (**b**) transverse velocity.

Additional velocity measurements were carried out with higher spatial resolution in the *y–z* plane to capture finer details of the wake flow dynamics. The measurements, conducted with a resolution of *0.2 mm *×* 0.27 mm*, were taken at *x* = *h*, *y* ∈ *[−h/2, h/2],* and covers one unit cell with *z* ∈ *[131.3,137]*. The results, depicted in Fig. 7, reveal a distinctive wake flow pattern characterized by a pronounced spatial periodicity. Analysis of the velocity components indicates that along the span, the wake flow alternates between jets with highly positive or highly negative transverse velocity, both with negligible streamwise velocity. This suggests that the free-stream is alternatively deviated from the streamwise direction towards the positive or negative transverse direction. These regions are separated by jets with high streamwise velocity and negligible transverse velocity forming an oscillating water sheet in the wake centerline. This highlights the structured nature and spatial periodicity of the wake flow shaped by the Gyroid architecture. Interestingly as illustrated by Fig. 5 and 6g,k, this flow pattern remains remarkably stable and persists into the far wake effectively inhibiting the formation and shedding of alternating vortices.

**Fig. 8:**
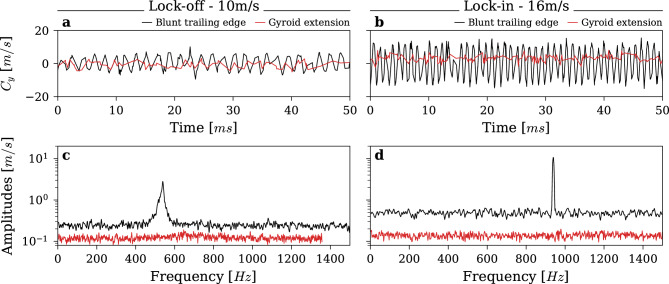
Flow transverse velocity measurement and its frequency analysis at steady velocity with the Blunt trailing edge and the Gyroid-based extension, at (**a**,**c**) C_ref_ = 10m/s. and (**b**,**d**) C_ref_ = 16m/s.

Benefiting from the high data rate of LDV, we can perform frequency analysis of the flow velocities to detect the presence of Kármán vortices. Figure 8 presents the transverse velocity signal and its spectrum in the wake center at *x* = *h*, at *C*_*ref*_ = *10 m/s* and *C*_*ref*_ = *16 m/s*. Spectra are computed using a method developed by Lenoir^32^, providing a method similar to Welch’s overlapped segment averaging for non-uniform sampled signals based on Lomb-Scargle periodograms^33,34^. In both conditions, the implementation of the gyroid extension results in a significant reduction in the fluctuations of the transverse velocity. Specifically, at *C*_*ref*_ = *10 m/s* the RMS value of the transverse velocity is reduced from *C*_*y,RMS*_ = *3.96 m/s* to *C*_*y,RMS*_ = *1.67 m/s*, and from *C*_*y,RMS*_ = *9.27 m/s* to *C*_*y,RMS*_ = *3.70 m/s* at *C*_*ref*_ = *16 m/s*.

Frequency analysis asserts the complete elimination of the vortex excitation with the gyroid extension. Respectively, at *C*_*ref*_ = *10 m/s* and *C*_*ref*_ = *16 m/s* the vortex shedding frequency is *f*_*s*_ = *540 Hz* and *f*_*s*_ = *938 Hz* resulting in a peak transverse velocity of *C*_*y*_ = *3.3 m/s* and *C*_*y*_ = *10.7 m/s* for the blunt trailing edge. The large increase under lock-in conditions is primarily attributed to the hydrodynamic coupling with the vibrating foil with only a minimal influence from the increase in freestream velocity. On the contrary, no distinct peak is observed at these frequencies with the gyroid extension, indicating the effective suppression of vortex shedding. Additionally, the overall noise level is significantly reduced with the gyroid extension compared to the blunt trailing edge. The uniform frequency response indicates that the very distinct wake pattern behind the gyroid extension shown in Fig. 7 is entirely composed of white noise, without any periodicity. The minor fluctuations shown in Figs. 5 and 6, are solely the result of shear mixing without temporal organization. And unlike a Kármán vortex street, this steady wake does not produce a periodic lift force and consequently, no vibration are generated.

### Wake flow revealed by cavitation

**Fig. 9:**
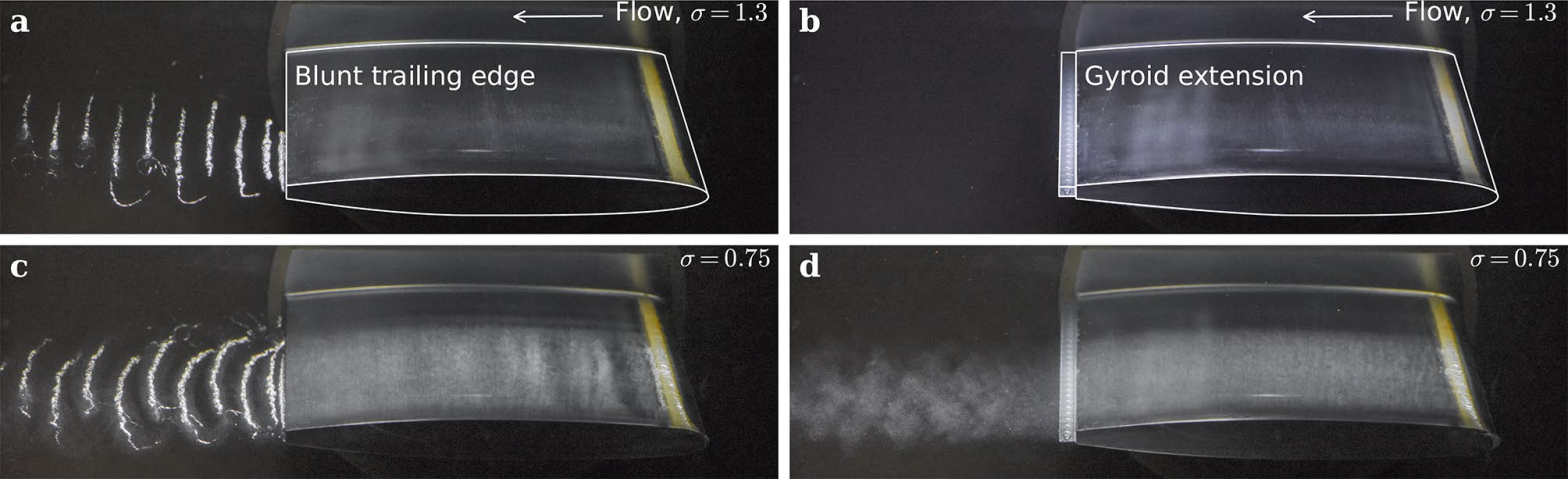
Side-views of the wake flow for C_ref_ = 16 m/s. (**a**,**c**) Baseline hydrofoil. (**b**,**d**) Gyroid-hydrofoil. (**a**,**b**) σ = 1.3. (**c**,**d**) σ = 0.75.

**Fig. 10:**
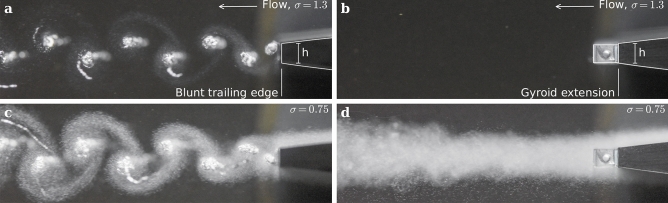
Close-up side-views of the wake flow for C_ref_ = 16 m/s. (**a**,**c**) Baseline hydrofoil. (**b**,**d**) Gyroid-hydrofoil. (**a**,**b**) σ = 1.3. (**c**,**d**) σ = 0.75.

To enable flow visualization of the wake, we lowered the pressure sufficiently to induce incipient cavitation, using the gas phase to trace the flow. Figures 9 and 10 present two side views captured at *σ* = *1.3* and *σ* = *0.75*, corresponding to cavitation inception in the wake of the baseline hydrofoil and at the leading edge of both hydrofoils, respectively. The upstream velocity is set to 16 m/s for both hydrofoils. At *σ* = *1.3*, cavitation develops within the core of the Kármán vortices for the baseline hydrofoil, while no cavitation is observed for the Gyroid hydrofoil. At *σ* = *0.75*, small cavitation forms at the leading edge of both hydrofoils, initiated by surface roughness. The resulting microbubbles travel downstream, becoming trapped in the Kármán vortices in the wake of the baseline hydrofoil, whereas no organized cavitation structures are observed in the wake of the Gyroid hydrofoil. This is a clear indication that the Gyroid insert remarkably prevents the formation of coherent structures and leads to a rather chaotic wake flow.

### Influence of angle of attacks

The analysis presented thus far has focused on the zero-incidence angle case. This configuration was deliberately chosen as it represents the most challenging scenario, where vortex-induced vibrations (VIV) and hydrodynamic lock-in are most pronounced. At 0°, the wake exhibits maximum vortex coherence, resulting in the highest vibration amplitudes and the strongest coupling between the vortex shedding frequency and the structure’s natural frequency.

To evaluate the robustness of the gyroid’s effect, we conducted additional tests by varying the incidence angle from − 5° to + 5°. Across this range, the gyroid-based trailing edge consistently demonstrated its ability to suppress vortex shedding and eliminate VIV, confirming its effectiveness under non-zero incidence conditions as well. This result is consistent with expectations, as the gyroid’s disruption of wake coherence is insensitive to moderate variations in flow angle.

**Fig. 11:**
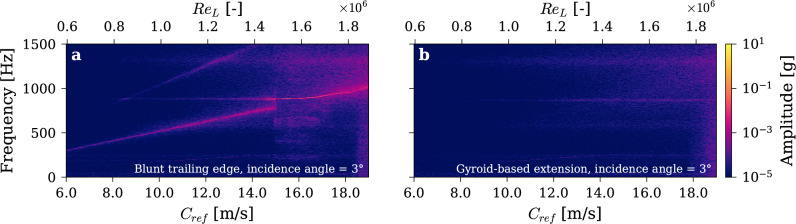
Time–frequency analysis of vibration signals during velocity ramp-up tests at 3 degree of angle of attack with (**a**) Blunt trailing edge and (**b**) Gyroid-based extension.

As a representative example, Fig. 11 presents the vibration response at an incidence angle of 3°. For the blunt trailing edge, clear VIV is observed, following a classical Strouhal relationship and exhibiting strong lock-in behavior when the shedding frequency approaches the hydrofoil’s torsional frequency. In contrast, the gyroid configuration shows no sign of lock-in or VIV, confirming its ability to maintain remarkable performance across a range of realistic incidence angles. Given the demonstrated robustness of vortex shedding suppression across varying incidence angles, we believe the gyroid’s effectiveness will extend to non-symmetric foils.

### Effects on hydrodynamic performances

**Fig. 12:**
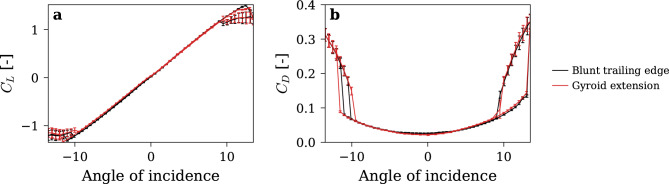
Performance of the hydrofoil with the Blunt trailing edge (in black) and Gyroid-based extension (in red). (**a**) Lift coefficient and (**b**) Drag coefficient, vs angle of attack.

The influence of the trailing-edge modification on the hydrofoil’s performance was evaluated to ensure that the substantial reduction in vibration did not come at the expense of overall hydrodynamic efficiency. To this end, lift and drag forces were measured and compared between the baseline and modified hydrofoils across a range of incidence angles, under a fixed freestream velocity of *C*_*ref*_ = *10 m/s*. As illustrated in Fig. 12, the lift and drag coefficients exhibit remarkable similarity between the two trailing-edge configurations. At low incidence angles, the modified design yields a slight reduction in drag, marginally enhancing performance. Conversely, at higher incidence angles, a minor decrease in lift is observed, leading to a subtle performance trade-off. At extreme angles of attack, both geometries demonstrate comparable stall onset and flow reattachment, indicating that the modification preserves the hydrodynamic performances of the hydrofoil while mitigating vibration.

## Methods

### Test facility, EPFL high-speed cavitation tunnel

The tests were carried out in the high-speed cavitation tunnel at EPFL, which features a *150 mm *× *150 mm *× *750 mm* test section. The facility supports a maximum freestream velocity of *50 m/s* and a maximum static pressure of *16 bar*.

The cavitation number *σ*, the Reynold number $\text{Re}$ and the Strouhal number *St*, associated with the shedding of Kàrman vortices, are defined as follows:

$$\sigma =\frac{{p}_{ref}-{p}_{v}(T)}{\frac{1}{2}\rho {{C}_{ref}}^{2}}, Re=\frac{{C}_{ref}L}{\nu }, St=\frac{{f}_{s}h}{{C}_{ref}}$$

where *p*_*ref*_ and *C*_*ref*_ are respectively the reference pressure and reference velocity, measured at the test section inlet. *ρ* is the water density and *p*_*v*_* (T)* the vapor pressure at water temperature *T*.

It should be noticed that the freestream velocity can be linearly increased/decreased at various acceleration rates. This way of operating the tunnel not only speeds up the tests but also ensures that no relevant event is missed, provided that the flow remains in quasi-steady state. For all subsequent tests, an acceleration of *0.085 m/s*^*2*^ was used, increasing the velocity from *6 to 20 m/s* over a duration of *160* s. For such a ramp-up, the relative change in velocity during one Strouhal period is ${{\dot{C}}_{ref} h}/{{C}_{ref}^{2} St} < 0.0042 \%$, supporting the quasi-steady hypothesis.

### Hydrofoils and gyroid based insert

The baseline hydrofoil is a rectangular planform stainless steel NACA 0009 with a span *B* = *149 mm*. The initial *110 mm* chord length is trimmed to *L* = *100 mm*, leaving a sharp blunt trailing edge of a thickness *h* = *3.22 mm*. The sharp edge ensures a consistent vortex shedding location, resulting in a Strouhal number that remains invariant across a broad range of velocities (*St ≈ 0.18*). The hydrofoil is mounted in the test section with a perfect embedding on one side while the other end is left free with a gap of *1 mm*. It is well known that the state of the boundary layer plays a major role in the wake dynamics. Our hydrofoils exhibit a hydraulically smooth surface for all tested velocities with a moving turbulence transition point. Therefore, the boundary layer is tripped at the leading edge with the help of a distributed roughness as described by Ausoni^29^.

The aim of the present study is to demonstrate how a specific porous insert, attached to the baseline hydrofoil trailing edge, can drastically reduce the vortex induced vibration. This study compares a 3D-printed porous gyroid-shaped extension with our baseline blunt geometry. The gyroid is approximated using a marching cube algorithm with the following level-set equation:

$$\varphi \left(x,y,z\right)=\text{sin}\left(\lambda x\right)\text{cos}\left(\lambda y\right)+\text{sin}\left(\lambda y\right)\text{cos}\left(\lambda z\right)+\text{sin}\left(\lambda z\right)\text{cos}\left(\lambda x\right)=0$$

where *λ* = *2π/C* and *C* corresponds the cubic unit cell (spatial period) in each direction. The final extension is generated using a unit cell *C* = *6 mm*, with *x* ∈ [2.5, 6.5] mm, *y* ∈ [−1.5, 1.5] mm, *z* ∈ [−75, 75] mm. The surface is then thickened and the proximal and distal faces are added using Blender^28^. The insert is then 3D printed with stereolithography Formlabs Form 3. The gyroid-shaped insert is finally glued to the baseline blunt trailing edge.

### Measuring apparatus

Vortex-induced vibrations are measured using a digital laser vibrometer (*Polytech, PDV-100*) on the hydrofoil surface at *(x/C, z/B)* = *(0.6, 0.5)* and an accelerometer (*KISTLER K-Shear 8702B)* mounted on the foil holder. Both signals were sampled at 20 kHz. Frequency analysis is performed using Welch’s overlapped segment averaging (WOSA) method over 60 segments of 1 s duration and an overlap of 50%. Spectrograms of the vibration signals are computed using short-time Fourier transforms with 1-s segments and 75% overlap.

The lift and drag coefficients are defined using the same chord length c, as the gyroid extension is extremely permeable and the added chord can be ignored.

$${C}_{L}=\frac{\text{Lift}}{0.5\uprho {C}_{ref}{A}_{ref}}, {C}_{D}=\frac{\text{Drag}}{0.5\uprho {C}_{ref}{A}_{ref}}, {A}_{ref}=L\times \text{B}=1.49\times {10}^{-2} {\text{m}}^{2}$$

The forces are measured using a 5-components load-cell^35,36^. The mean value of the lift and drag coefficients was measured during 2 s, sampled at 1000 Hz in a non-cavitating flow under lock-off conditions *Re* = *10*^*6*^ ± *5%.*

Velocity field measurements were performed with help of a *Dantec Flow Explorer* single-point 2-components Laser Doppler Velocimetry (LDV) with 10 μm hollow glass spheres as seeding particles. The frequency analysis of the randomly sampled LDV signal was performed using Lomb-Scargle periodograms^33,34^. This approach, which is largely used in astronomy, allows efficient and accurate computation of frequency spectrum with non-uniform sampling. To reduce the variance of the periodograms estimation, a method similar to the Welch’s overlapped segment averaging (WOSA) developed by Lenoir^32^, was applied.

## Conclusion

In this study, we discovered the exceptional potential of a novel flow control approach, based on Gyroid structures. We demonstrated that a Gyroid-based extension attached to the trailing edge of a blunt NACA 0009 hydrofoil remarkably prevents the roll-up and shedding of alternate vortices and effectively reduces induced vibrations across a wide range of Reynolds numbers (*Re* = *0.6* × *10*^*6*^* to 2* × *10*^*6*^). The key findings may be summarized as follows:

- *Complete suppression of vortex shedding*: The Gyroid-based extension achieves the complete elimination of vortex shedding. The wake exhibits a broadband frequency spectrum with no discernible frequency peaks across the entire tested velocity range.
- *Stabilized wake*: The wake behind the Gyroid trailing edge displays a distinct periodic pattern, with alternating transverse and streamwise jets, matching the spanwise periodicity of the Gyroid insert. This pattern, characterized by random and low amplitude fluctuations, persists even in the far wake, and its remarkable streamwise stability appears to play a crucial role in the suppression of vortex shedding.
- *No more lock-in*: By eliminating vortex-induced excitation, the hydrofoil experiences at least 67% reduction in vibrations. Notably, the lock-in hydrodynamic coupling, which typically leads to a sharp increase in vibration amplitudes, is entirely eradicated with the Gyroid trailing edge, resulting in a remarkable 99.5% reduction in vibrations under lock-in conditions.
- *No alteration of hydrodynamic performance*: The hydrofoil’s lift and drag remain almost unchanged at both low and high angles of attack.

## Data Availability

Data generated during the current study are available from the corresponding author upon reasonable request.
